# Increasing familial engagement in family violence services: a systematically conducted realist review of barriers and facilitators

**DOI:** 10.3389/fpsyt.2026.1588381

**Published:** 2026-07-08

**Authors:** Stefan Kurbatfinski, Julia Wilson, Daniel Cosic, Alexa Toews, K. Alix Hayden, Sarah Erickson, Nicole Letourneau

**Affiliations:** 1 Department of Community Health Sciences, Cumming School of Medicine, University of Calgary, Calgary, AB, Canada; 2 Owerko Centre, Alberta Children’s Hospital Research Institute, Calgary, AB, Canada; 3 Wood PLC Vibrations, Dynamics, and Noise, Calgary, AB, Canada; 4 Libraries and Cultural Resources, University of Calgary, Calgary, AB, Canada; 5 Department of Pediatrics, Cumming School of Medicine, University of Calgary, Calgary, AB, Canada; 6 Department of Psychiatry, Cumming School of Medicine, University of Calgary, Calgary, AB, Canada; 7 Faculty of Nursing, University of Calgary, Calgary, AB, Canada

**Keywords:** abuse, barriers and facilitative factors, family violence, service engagement, strategy, support

## Abstract

**Purpose:**

Physical, emotional, and sexual violence and abuse occurring between close or extended family members are forms of family violence (FV). The negative impacts of FV are diverse, numerous, and pervasive for all individuals involved. To effectively support individuals experiencing FV, services designed to reduce and prevent FV and its associated harms must maintain participant engagement. Therefore, the purpose of this realist review was to synthesize literature on barriers and facilitators to individuals’ engagement in FV services so that relevant strategies can be implemented to improve service effectiveness.

**Methods:**

This realist review was conducted systematically and searched five databases up to August 2024, screening 1, 334 evidence sources. Findings were examined in relation to families and parents, including mothers, fathers, and children, and were supplemented by relevant service provider perspectives.

**Results:**

Most findings from the 24 included studies focused on facilitators of engagement. Priming and introductory sessions, provider-family rapport, group formats, empathy, trustworthiness, and family-centred approaches that consider familial needs and goals were identified as important facilitators of engagement in FV services. Prominent barriers included socioeconomic challenges, competing demands, childcare responsibilities, inadequate provider-client rapport, limited accessibility, intense initial meetings, and long service duration.

**Conclusions:**

Rooted in family-centred approaches and interagency collaboration, recommended strategies to improve family engagement in FV services focus on opportunities during service: 1) intake; 2) initiation; and 3) delivery. These strategies are described in relation to context (e.g., socioeconomic factors) and potential mechanisms (e.g., perceived cultural safety), and could be further evaluated using implementation science methods to optimize family engagement in FV services.

## Introduction

Physical, emotional, and sexual violence, as well as other forms of violence (e.g., identity-based, religious) occurring between close or extended family members, intimate partners, or parents and children (including child maltreatment such as abuse and neglect) are forms of family violence (FV) (1). The negative impacts of FV are diverse, numerous, and pervasive, negatively impacting everyone involved (2), particularly mothers (3, 4) and their children (5, 6). Although fathers are also negatively affected and are often recognized as part of the solution (7), engaging them in FV-related services remains challenging (8). Services that address FV, such as home visitation, training, or counselling programs (9, 10) are essential for reducing and preventing the negative impacts of FV on quality of life (11, 12). Research demonstrates that these services can reduce the occurrence of FV when participants actively attend and complete them (13). However, such services may be ineffective if participants do not enroll, drop out, or disengage before achieving treatment goals (13). Therefore, a comprehensive review of literature examining barriers and facilitators to family engagement in FV services is essential. This knowledge may help identify strategies that encourage families to remain actively engaged throughout relevant services and maximize potential benefits.

### FV prevalence and disclosure

All family members are affected by FV. Findings from a systematic review indicated that, globally, more than 50% of children aged 2 to 17 years old, approximating one billion children (or 14% of the world’s population), experience child abuse annually (14). Among women aged 15 to 49 years old, the global lifetime prevalence of physical and/or sexual intimate partner violence (IPV) is estimated at approximately 27%, with higher rates in low-income countries compared to high-income countries (15). Among men, global prevalence estimates for experiencing violence range from 7.3% to 37% for psychological violence, 3.4% to 20.3% for physical violence, and 0.2% to 7% for sexual violence; however, most of these men had perpetrated violence themselves (16). While prevalence of violence may be similar across groups, women are more likely to be seriously injured, poly-victimized, and fearful of being killed during violent exchanges with intimate partners (17, 18). Recently, COVID-19-related public health policy changes revealed repercussions of social isolation on FV occurrence, with police-reported data showing no change in FV occurrence while community-based data revealed an increase (19). These findings suggest that individuals experiencing FV may have been less likely to access formal supports despite increasing occurrence, thereby limiting the acquisition of relevant care.

Although FV prevalence is high, it is likely underreported because of coercive control, the cycle of abuse, and other vulnerabilities (20). Family members experiencing coercive control (e.g., being monitored, financially controlled, isolated) may experience confusion, fear, and stigma as they become increasingly dependent on the abusive partner (21, 22). Coercive control is closely linked to the cycle of abuse, in which abusive behaviours are often followed by apologetic or reparative actions intended to restore the relationship (23). When successful, the abusive partner often engages in increasingly severe aggressive and apologetic behaviours, escalating the cyclic nature of abuse (23). Further, children are challenged by their ability to report FV as they: 1) may not conceptualize their experiences as abuse, 2) may be unable to access services independently (e.g., no transportation), or 3) may require external support (e.g., teachers) to report their experiences (24, 25). In addition, mandatory reporting laws can often discourage honest engagement in FV services, as parents may fear incarceration of their partner (26), family dissolution, or involvement of child welfare services leading to the removal of their child(ren) (21). Although mandatory reporting is necessary in some instances, there must be flexibility in policy design and service delivery, as rigid approaches can unintentionally worsen quality of life when implemented without sufficient justification.

### Impacts and costs of FV

Those who do not seek or receive support for their experiences of FV are at a greater risk of enduring negative impacts (20). FV can result in injuries (e.g., fractures, head and neck trauma, bruising) (22), hospitalization, and even death (2), as well as emotional consequences including fear, shame, embarrassment, isolation, and grief (21, 27). Those who escape violence are also at a heightened risk of later developing mental health conditions such as anxiety, depression, and substance use disorders (20, 28). In addition to adverse health outcomes, FV increases the likelihood of unemployment, lower income (29), homelessness (30), and suboptimal educational performance (31, 32), all of which are associated with reduced quality of life.

Not only does FV result in various impacts that decrease quality of life globally (33), but it has also been estimated to cost the global economy at least 8% of the gross domestic product (34, 35). Associated healthcare, legislative, social service, and labour productivity costs are substantial (36). A study conducted in the United States estimated that violence against women and men cost approximately USD $3.3 trillion and $281 billion, respectively, across the lifespan, for a total of $3.6 trillion (37). Another study estimated violence against children costs USD $7 trillion globally each year (35). Investing in FV services can simultaneously reduce both the impacts of FV on quality of life and the associated economic burden.

### FV intervention services

Many services have been designed to support families experiencing FV. Home visitation services can include parental counselling, support, and education (10), such as “listening” visits that promote mental health (38) and parent-child interaction guidance to foster healthier family dynamics and reduce FV (39). Findings demonstrate that home visitation services generally reduce child maltreatment (10), while also reducing violence between partners, at least in the short-term (40). Other services, such as those focused on substance use and mental health conditions (41), sibling abuse (42), and socioeconomic concerns (43) also seem promising in reducing FV. However, batterer intervention services, which are designed to reduce abusive partners’ violent behaviours through education, shared experiences, and cognitive reframing, appear to be less effective (9). Regardless of service type, the effectiveness of FV service is ultimately undermined by suboptimal engagement among enrolled participants (44–47). Understanding the barriers and facilitators to engagement could help inform more effective strategies that promote higher engagement, and, ultimately, better outcomes for family members experiencing or engaging in FV.

### Barriers and facilitators to engagement in FV services

Although families may initially enroll in FV services, sustained engagement can be hindered by numerous factors, in addition to stigma, discrimination, and the personal values of families and service providers (23, 27, 48). FV is often compounded by broader familial concerns such as financial instability, employment challenges, neighbourhood socioeconomic status, cultural understandings, substance use disorders, educational attainment, and intergenerational trauma, among others (20, 49). These factors can serve as competing priorities that challenge families’ engagement in FV services (50). Individuals identifying with minority groups (e.g., racial, sexual, or gender minority) may also face additional social forces such as discrimination, myths, and stigmas that affect help-seeking behaviours (48, 51).

Conversely, several factors appear to facilitate engagement in FV services. Warm, optimistic, and supportive responses from service providers have been shown to promote engagement in FV services (26, 48, 52). For example, stronger provider-client rapport in FV services targeting men has been linked to decreased violence within relationships (53). Further, group-based formats can promote engagement in FV services by fostering a collective experience within a destigmatized and safe environment when implemented appropriately (54). FV services that effectively reduce barriers and promote facilitators are expected to result in greater levels of family engagement.

### Purpose of the review

To our knowledge, a review on barriers and facilitators that enhance individuals’ engagement in FV services has not been conducted. Therefore, this realist review, conducted systematically, sought to: 1) synthesize literature on barriers and facilitators to families’ engagement in FV services and 2) recommend strategies based on relevant findings that may elicit greater engagement in FV services.

## Materials and methods

Through realist review methods (55), barriers and facilitators to engaging families in FV services were identified. The realist review approach, conducted systematically, enabled the pragmatic determination of barriers and facilitators to families’ engagement in FV services. These findings were first synthesized descriptively and subsequently interpreted through a realist lens in the discussion to generate potential strategies to increase engagement in FV services based on context and potential mechanisms. Further, this approach allowed for the recommendation of relevant strategies for service providers to elicit greater FV service engagement (55, 56). As no primary data were collected, ethics approval was not needed.

### Search strategy and inclusion criteria

Included studies: 1) were peer-reviewed; 2) written in English; 3) included a family structure composed of at least one child, and mother(s) and/or father(s); 4) described at least one barrier and/or facilitator that impacted families’ engagement in FV services; 5) did not involve medical interventions; 6) were conducted in any geographic location; 7) included any participant demographic; 8) could be from the perspective of family members experiencing or engaging in FV or their service providers; and 9) could be quantitative, qualitative, or mixed-method study designs. We excluded studies that examined women or men experiencing FV, including IPV, if the individuals were not parents or caregivers, non-peer reviewed literature, grey literature, and studies focused on preventive strategies of FV in which FV had not yet occurred.

APA PsycINFO (OVID) was searched prior to modifying the search for other databases, including CINAHL Plus with Full Text (Ebsco), SocINDEX with Full Text (Ebsco), Family & Society Studies Worldwide (Ebsco), and Social Work Abstracts (Ebsco). The search strategy was guided by an expert health sciences librarian (KAH) and were translated and ran by two authors (JW, DC). The search strategy consisted of the following concepts: family (i.e., parents, caregivers, youth), engagement (i.e., engage, participate), violence (i.e., neglect, physical, sexual, or emotional abuse), and intervention (i.e., programs, services). Keywords and subject headings were adapted according to the database indexing. All search strategies are provided (see **Supplementary Tables A–E**).

**Figure 1:**
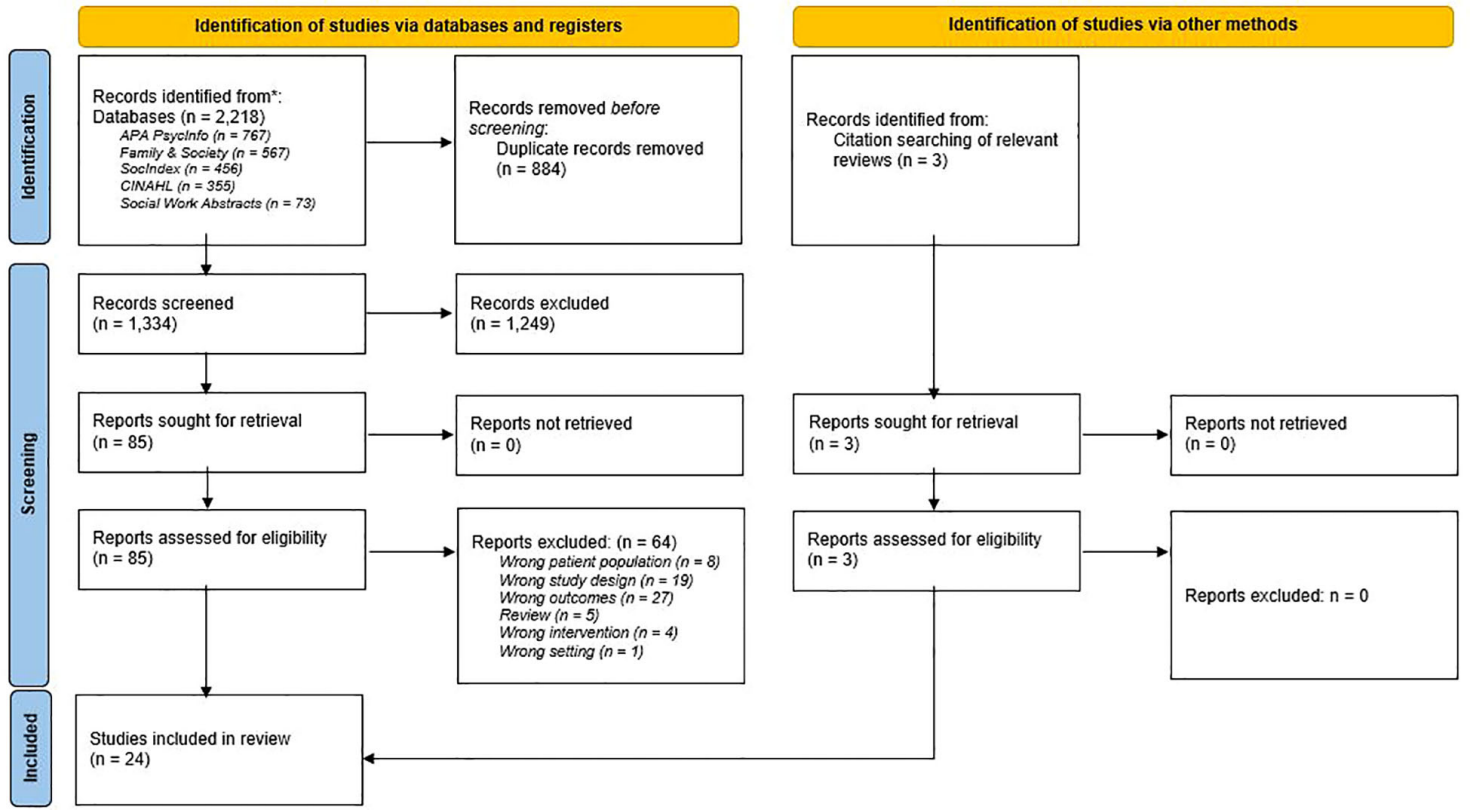
PRISMA flow diagram.

All the searches were conducted August 13^th^, 2024. Retrieved records (n=2,218) were uploaded onto Covidence (57), a web platform designed for conducting reviews that facilitates data organization, screening, and extraction. Four individuals (SK, JW, DC, LM) completed abstract and title screening; all reviewers completed pilot screening of 50 random evidence sources and obtained over 90% percent agreement. After manual and auto removal of duplicates (n=884), 1,334 evidence sources were screened during abstract and title review, identifying 85 studies for full-text review. Five individuals (SK, JW, CP, DC, NL) screened the full texts using the same inclusion criteria. Of the 85 studies that were screened, 21 were included for data extraction, although an additional 3 studies were found after reference mining relevant reviews. A PRISMA flow diagram is provided to visualize the screening process along with reasons for full-text exclusion (**Figure 1**).

### Data extraction and synthesis

Three authors (DC, LM, AT) created and piloted a form to extract, organize, and synthesize data and findings. Three authors (SK, JW, DC) extracted relevant study information (i.e., study design, demographic composition, country of origin, sample size) to characterize the included studies. Findings were synthesized iteratively to identify patterns explaining how barriers and facilitators influenced engagement in FV across diverse contexts and populations. Although findings were initially organized according to barriers and facilitators among target groups (i.e., families/parents, mothers, fathers, children), including service provider perspectives when data were available, interpretation also considered contextual conditions and potential underlying mechanisms that may help to explain engagement outcomes in FV services. This realist-informed approach aimed to better understand what works, for whom, and under what conditions. These explanatory patterns informed the development of recommendations and the conceptual model presented later in the Discussion section. Formal quality appraisal was not undertaken because realist reviews prioritize the contribution of evidence toward explanatory understanding and theory development, rather than focusing on study quality scores.

## Results

Of included studies (n=24), most were conducted in the United States (n=11), followed by Australia (n=6), Sweden (n=2), the United Kingdom (n=1), Ireland (n=1), Lebanon (n=1), China (n=1), and one study which used multiple regions (i.e., North Macedonia, the Republic of Moldova, Romania). The most frequent study designs were qualitative (n=13), followed by randomized controlled trials (n=5), quasi-experimental (n=2), mixed-method (n=2), and program evaluations (n=2). Information about included studies is provided (**Supplementary Table F**).

### Barriers to family, mother, and father engagement in FV services

**Figure 2:**
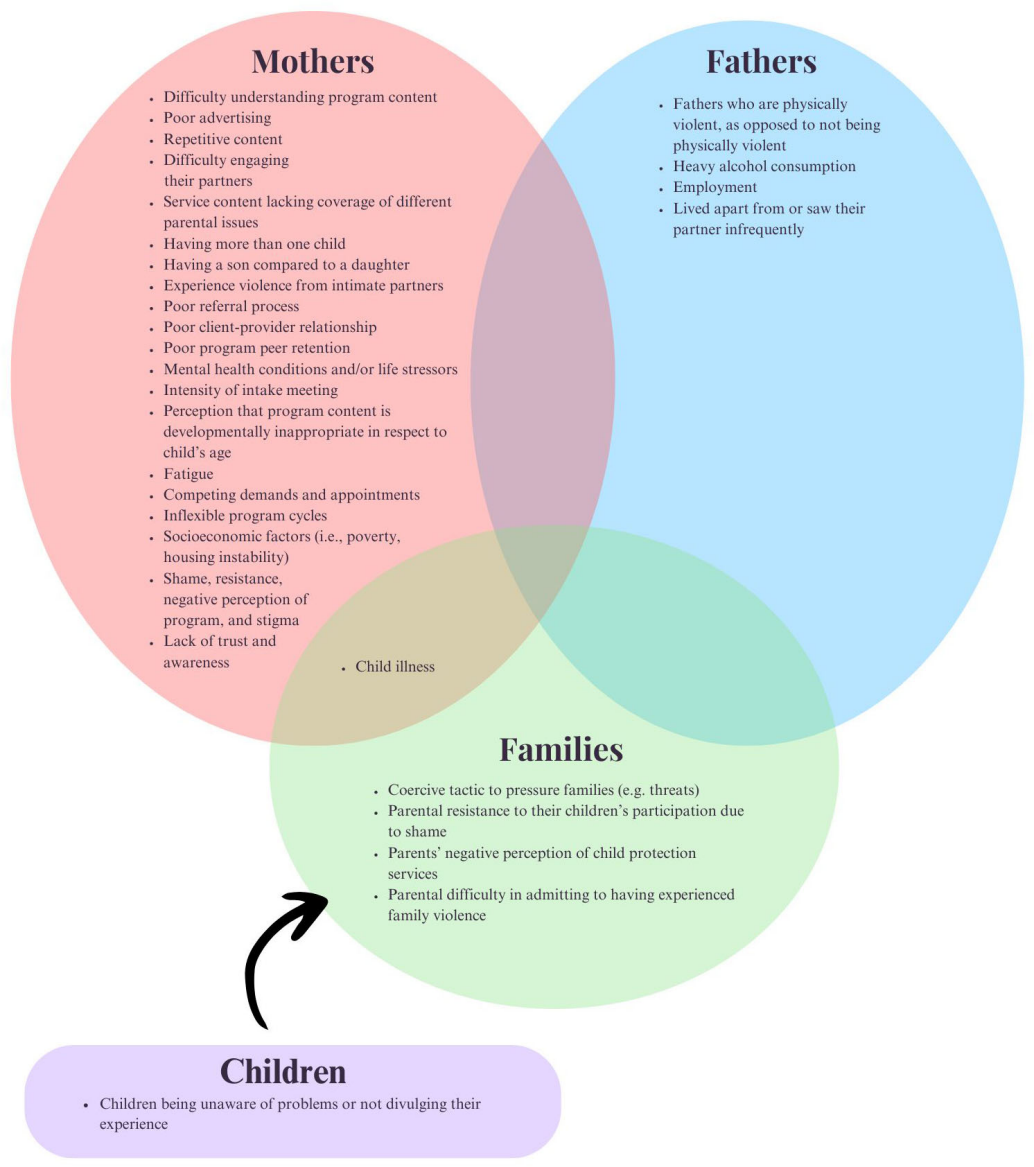
Venn diagram comparing barriers to engagement for mothers, fathers, families, and children.

Eight studies described barriers to family, parent, and child engagement in FV services. Of these, two evaluated barriers to engagement from the perspectives of child protection service providers, while six examined barriers from the perspectives of family members. A visual comparison of identified barriers is provided in a Venn diagram (**Figure 2**).

#### Barriers to families’ engagement

*Service Provider Perspectives.* Two studies examined barriers to families’ engagement in FV services. Both studies qualitatively gathered service provider perspectives on barriers affecting parents’ and children’s engagement in FV services. Professionals working in child protection services identified service provider judgement and the use of coercive tactics intended to pressure families (e.g., threats, shaming behaviours) as barriers to engagement (58). Svensson et al. (59) identified three additional barriers to engagement in FV services using qualitatively collected perspectives from child protection service support group leaders: 1) parental resistance to children’s engagement due to shame; 2) parents’ perception of child protection services; and 3) parents’ difficulty admitting experiences of FV.

#### Barriers to mothers’ engagement

Five studies examined barriers to mothers’ engagement in services from the perspectives of parents.

*Parental Perspectives.* Leckey et al. (60) qualitatively described mothers’ barriers to engagement in a wraparound intervention that integrated various parenting programmes, called the Children At Risk Model. Identified barriers included difficulty in understanding service content, beliefs that the content was developmentally inappropriate for their older children, poor advertising, repetitive content, other peer members leaving the program, poverty, housing instability, social isolation, fear, stigma, and mental health conditions (60).

In relation to home visiting services, Fogarty et al. (61) provided insight on barriers to parental engagement in the Home Parenting Education support services (HoPES) through interviews with mothers. Identified barriers included life stress, child illness, fear of judgment, competing demands, the transition to parenthood, difficulty engaging partners when mothers wanted their involvement, stress and fatigue, limited coverage of different parenting issues, the intensity of intake meetings, lack of trust, lack of awareness, inflexible service cycles, poor referral processes, and overall service duration (61). Using qualitative data, Balgopal et al. (62) also identified child illness as a barrier, whereas Williams et al. (63) identified service intensity and lack of childcare as barriers to maternal engagement. Both studies also identified scheduling conflicts as a factor that impeded service engagement (62, 63). In the study of 140 parents (137 mothers; 75% completion rate), experiencing intimate partner violence was also identified as a barrier to mothers’ engagement in FV services that are designed to reduce child abuse and neglect (63).

Lastly, using a mixed-methods study design, Farrell et al. (64) assessed the Supportive Housing for Families Programme among 41 parents (40 mothers), which was designed to provide immediate housing support to mothers experiencing housing and child welfare challenges. Lack of understanding of the provider’s role and poorer provider-client rapport resulted in lower parent engagement scores (mean score below 5.3) on the Parent Engagement Measure (65) compared to those who reported a stronger relationship with their caseworker (mean score above 5.9) (64).

#### Barriers to fathers’ engagement

*Parental Perspectives.* One study identified barriers to fathers’ engagement in FV services. In this randomized controlled trial, Duggan et al. (66) examined violent and non-violent fathers’ engagement in home visitation services and found that only half of participating families remained engaged after one year, reflecting significant attrition (66). Fathers who were physically violent, consumed alcohol heavily, were employed (e.g., difficulty coordinating service delivery time), or lived apart from or saw their partner infrequently were less likely to engage in home visitation services (66).

#### Barriers to children’s engagement

*Service Provider Perspectives.* Only one study explicity reported on barriers to children’s engagement in FV services. Svensson et al. (59) indicated that children being unaware of FV-related problems or withholding their experiences undermined active engagement in FV services.

### Facilitators to family, mother, father, and child engagement in FV services

Twenty-two total studies examined facilitators to FV service engagement among families and parents, including mothers, fathers, and children. Findings are derived from various intervention modalities and study designs, with seven studies describing service provider perspectives, including those of home visitors (n=1), social workers (n=2), child protection service providers (n=3), and counsellors (n=1).

#### Facilitators to families’ engagement

**Figure 3:**
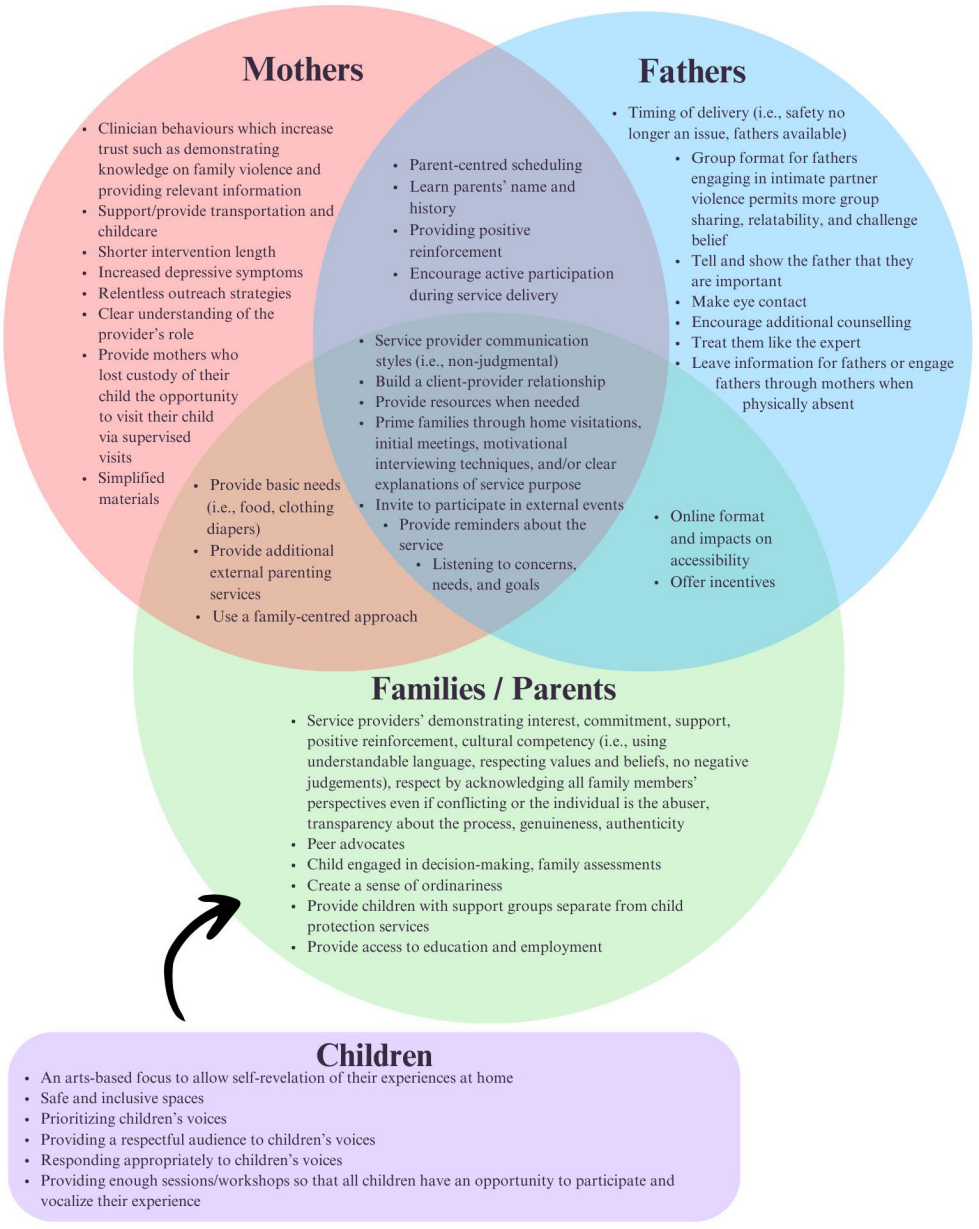
Venn diagram comparing facilitators to engagement for mothers, fathers, families, and children.

Ten studies examined facilitators to family engagement in FV services. These studies used either parental (n=4), service provider (n=5; 3 child protection service providers, 1 social workers, 1 home visitors), or both (n=1) perspectives to evaluate factors that enable parental engagement in FV services. A visual comparison of facilitators is provided in a Venn diagram (**Figure 3**).

*Parental Perspectives.* Strolin-Goltzman et al. (67) examined the impact of an online version of The Breakthrough Parenting Curriculum among parents involved with the child welfare system. Participants demonstrated high completion rates (72%), which appeared to be attributed to the accessibility and no-cost nature of the online intervention, although this was not explicitly analyzed or drawn from participants’ perspectives (67). In a qualitative evaluation of a parenting training service (62), the use of home visitations conducted before and throughout the training program appeared to prime parents and increase their engagement (83% attendance rate; 6% dropout rate). Parents identified service providers’ interest, commitment, support, and positive reinforcement during home visitations as key facilitators to engagement (62).

Chaffin et al. (68) used a randomized controlled trial to compare a self-motivational orientation with a standard informational orientation among parents involved with child welfare services due to child physical abuse and/or neglect. The self-motivational orientation exposed parents to successful and motivating parenting stories and encouraged them to identify their current problems, reflect on goals, and commit to changing their behaviour (68). This approach resulted in higher retention rates (85%) compared to the standard informational orientation (56%) (68), and 100% retention was observed among parents who had reported low-to-moderate motivation prior to receiving the motivational orientation.

In another randomized controlled trial comparing the SafeCare1 home visitation service with Services as Usual among culturally diverse families, Damashek et al. (69) evaluated approaches to engagement measured by family satisfaction and goal completion. Participants’ engagement was fostered by service providers who demonstrated greater cultural competency (69). Providers who respected cultural differences (i.e., used understandable language, respected cultural values and beliefs, avoided negative judgment) and considered the broader family and community (i.e., allowing familial autonomy in decision-making and tracking progress, using community and family supports) fostered greater participant engagement (69).

*Service Provider Perspectives.* Healy and Darlington (70) used a qualitative case study vignette to examine child welfare service staff’s strategies for engaging families. They identified three core principles: 1) respect, including acknowledging each family member’s perspectives, knowledge, and opinions, including the child, even when contributing to FV; 2) appropriateness, referring to appropriate family assessments, rapport-building, and including children in decision-making; and 3) transparency, emphasizing openness regarding the strengths and weaknesses of involving children and parents throughout the process. Service providers in a study conducted by Seekamp et al. (58) paralleled these findings, but also emphasized the importance of collaborating with other service agencies. Meanwhile, social workers in another study focused on timing, indicating that connecting quickly with family members after enrollment facilitated engagement (71). In another qualitative study, child welfare service staff identified four approaches to destigmatizing service provision: 1) balancing normalization of the family situation while confirming the children’s needs; 2) preparing children and parents through initial meetings; 3) creating a sense of ordinariness; and 4) using deliberate recruitment strategies (i.e., marketing through flyers, adverts, social media posts, webpages, and networking events) that separate support groups from child welfare services (59). Bellamy et al. (72) reported similar findings in their qualitative study and additionally identified several facilitators to parental engagement in FV services, including: 1) supporting socioeconomic needs such as education, employment, and housing; 2) asking consistent questions, differing only when parents’ needs or attendance varies; 3) inviting families to events outside of direct FV service; 4) providing service reminders; 5) scheduling around parents’ commitments; and 6) offering incentives for engagement.

*Both Parental and Service Provider Perspectives.* Fitz-Symond et al. (73) qualitatively described a peer parental advocacy service in which supervised peer advocates with lived experience were assigned to families involved in child welfare services with three interventive stages: 1) pre-advocate involvement, where peer advocates were matched to families based on their multifaceted needs; 2) advocate involvement, where peer advocates provided emotional support, guidance, and connections to professionals; and 3) ongoing support, where peer advocates continued to ensure families felt heard (73). These stages appeared to promote parental engagement in child welfare services by helping parents navigate mistrust, power imbalances, emotional challenges, and the complex language and processes associated with the child welfare system (73).

#### Facilitators to mothers’ engagement

Seven studies examined facilitators to mothers’ engagement in FV services using mothers’ (n=6) and service providers’ perspectives (n=1; home visitors).

*Parental Perspectives.* In their qualitative study using semi-structured interviews, Fogarty et al. (61) reported that clinician behaviours, communication styles, and characteristics were important facilitators of engagement. Specifically, clinicians who demonstrated knowledge and provided relevant information increased mothers’ trust (61). Non-judgemental communication that normalized familial challenges increased mothers’ comfort during home visits, encouraged them to ask questions, and reduced perceived threats (61). Additionally, following through on mothers’ requests strengthened service provider reliability, treatment relevance, therapeutic relationships, parent cognitions and beliefs about treatment, and overall service delivery (61). In addition to provider characteristics, weekly reminders, transportation, and the provision of childcare were identified as factors that increased mothers’ engagement in a mixed-methods study focused on a group training service (63). Moreover, strong provider-mother rapport and shorter intervention length were identified as significant predictors of higher service completion in a randomized controlled trial focused on Families Connection, a community-based service that aims to identify and fulfill familial needs (98% of sample was mothers) (74). Greater maternal depressive symptoms also predicted increased FV service engagement, particularly service completion (74).

Chablani and Spinney (75) applied a more interdisciplinary approach in their Circle of Care program evaluation, involving an amalgamation of home visiting, motivational interviewing, educational programming, and support groups. A strategy described as “relentless outreach” was used to engage high-risk young mothers at a high retention rate (90%), consisting of continuous outreach attempts even when mothers fell below program guidelines for attendance, along with strong provider-mother rapport based on trust and family-oriented needs (75). In a mixed-methods study of the Supportive Housing for Families Programme, mothers’ engagement increased when accessibility to and applicability of a caseworker were enhanced (64). In addition to provider-mother rapport, qualitative and quantitative findings revealed that mothers were more engaged when caseworkers were resourceful, responsive, and effectively explained their role (64).

A culturally adapted Parenting Through Change for Reunification (PTC-R) service engaged parents (13 of 14 female) involved with child welfare services in a group training service (76). Qualitatively, identified facilitators to engagement included: 1) introductory sessions focused on explaining the intervention, fostering hope, and addressing negative emotions experienced by previous participants; 2) opportunities for parent-child visitation to practice parenting and skill-building after losing custody of their children; 3) simplifying intervention materials; and 4) modifying examples and role-plays to address cultural and contextual differences (76). More specifically, interventionists adapted language to challenge normalized perceptions of FV and increase parents’ understanding of its implications (76).

*Service Provider Perspectives.* Bellamy et al. (72) reported qualitative findings which paralleled previous studies and identified several additional facilitators to mothers’ engagement, including: 1) providing families with basic necessities including diapers, clothing, and breastfeeding resources; 2) intentionally involving mothers and asking them to engage in activities; 3) clearly explaining the purpose of services; 4) providing positive reinforcement; and 5) using reminder calls and text messages.

#### Facilitators to fathers’ engagement

Seven studies evaluated facilitators that improved violent/abusive fathers’ engagement in FV services. Of these, four used perspectives from service providers (i.e., 1 home visitors, 1 counselors, 2 social workers) and three used perspectives of participating fathers.

*Parental Perspectives.* Guterman et al. (77) examined the Dads Matter-HV program, a home visitation service for fathers, through a randomized controlled trial with an 84% retention rate at 12 months post-enrollment. Timing was identified as an important facilitator to fathers’ engagement in home visitation services, whereby it was important to engage fathers when it was more practical to do so (e.g., safety issues are no longer a concern and fathers are in fact available) (77). Further, initiating Dads Matter-HV postnatally, rather than prenatally, was a “magic moment” that promoted paternal engagement.^77(p11)^ Postnatal initiation improved provider-father rapport, which subsequently increased paternal support toward mother and reduced child abuse (77). In addition to timing, service content was individualized to address specific family needs while remaining within available service resources (77).

Further, one quasi-experimental study evaluated a group-based intervention aimed at reducing fathers’ abusive behaviours towards partners and reported a completion rate of 82.4% (78). Group sessions enabled male abusers to share their stories and stressors, challenge their relationship beliefs, and develop awareness and relational skills, thereby increasing engagement in the service (78). Participants described the setting as supportive and useful, with trust increasing over time and low dropout rates attributable to the group format (78). The effectiveness of group formats in engaging fathers was also demonstrated within different cultural groups (i.e., Syrian and Lebanese individuals) for whom FV and mental health are taboo topics (79).

*Service Provider Perspectives.* Bellamy et al. (72) identified numerous facilitators that increased fathers’ engagement in FV services, including: 1) clearly explaining the potential impact of services on the father-child relationship; 2) emphasizing fathers’ importance and role in their children’s developmental trajectory; 3) intentionally involving fathers by asking about their needs, concerns, and parenting goals; 4) inquiring specifically about employment needs and goals; 5) treating them like the expert; 6) encouraging fathers to participate in activities; 7) using online platforms when fathers are physically absent; 8) inviting fathers to activities outside of the direct services (e.g., family dances, father-child events); 9) focusing on rapport-building and comfort; 10) leaving information for fathers or engaging them indirectly through mothers when physically absent; and 11) using incentives.

In a study conducted among counsellors, five facilitators were identified when working with men engaging in abusive or violent behaviours (80). This included: 1) ‘noticing and curiosity,’ which involved acknowledging FV and encouraging fathers’ to self-identify abusive behaviours; 2) ‘balancing,’ which focused on discussing gender constructs while being attentive to both the experiences of the person engaging in FV and the person experiencing FV; 3) ‘focusing on him for her,’ which encouraged men to seek counselling to better support their female partners, contextualizing discourse on ethics and power’; 4) ‘power and control,’ which examined partner interactions in a safe space to identify power and control tactics; and 5) ‘possibilities,’ which encouraged exploration of alternatives to FV within relationships (80). Such findings were echoed by social workers through qualitative responses which emphasized the importance of creating trusting bonds with families (71, 81). More specifically, social workers described minimizing tension, avoiding confrontation, and supporting and affirming fathers while addressing the abusive behaviour as facilitators to engaging fathers (81).

#### Facilitators to children’s engagement

Two studies evaluated facilitators that increased children’s engagement in FV services. Of these, one used perspectives from children and one used perspectives from social workers.

*Children’s Perspectives.* One study used music workshops to engage children (n=15) aged 8 to 14 years who were experiencing vulnerabilities or FV through a child-centered approach (82). This workshop was facilitated by fostering safe and inclusive spaces for children and prioritizing their voices through respectful listening and appropriate responses (82). Providing a sufficient number of workshops was also important to ensure that all children had opportunities to share their experiences (82).

*Service Provider Perspectives.* Service providers also qualitatively described the implementation of support groups for children that are separate from child welfare services to improve coping strategies and promote resilience (59). Specifically, creating a sense of ordinariness and incorporating creative activities such as drawing and play supported children’s engagement in FV support groups (59).

## Discussion

This realist review appears to be the first of its kind, systematically identifying barriers and facilitators that can inform effective strategies to increase attendance and active engagement of families in FV services. Of the 24 included studies, most focused on barriers and facilitators related to engaging families or parents collectively, rather than specifically examining mothers, fathers, or children independently. Overall, findings highlight several service provider characteristics, attitudes, and behaviours that facilitated engagement in FV services, including showing interest in families, cultural competency, and taking time to develop rapport. Promoting accessibility (e.g., addressing socioeconomic concerns, online formats), adopting family-centred care in community-based FV services (83), and implementing priming approaches such as introductory sessions were also identified as important facilitators to FV service engagement. In addition, each group demonstrated barriers and facilitators that were unique to their specific experiences and needs. Using a realist review approach, the discussion focuses on interpreting the identified barriers and facilitators to understand specific contextual factors and potential mechanisms that may influence engagement outcomes in FV services.

### Enhancing family engagement

**Figure 4:**
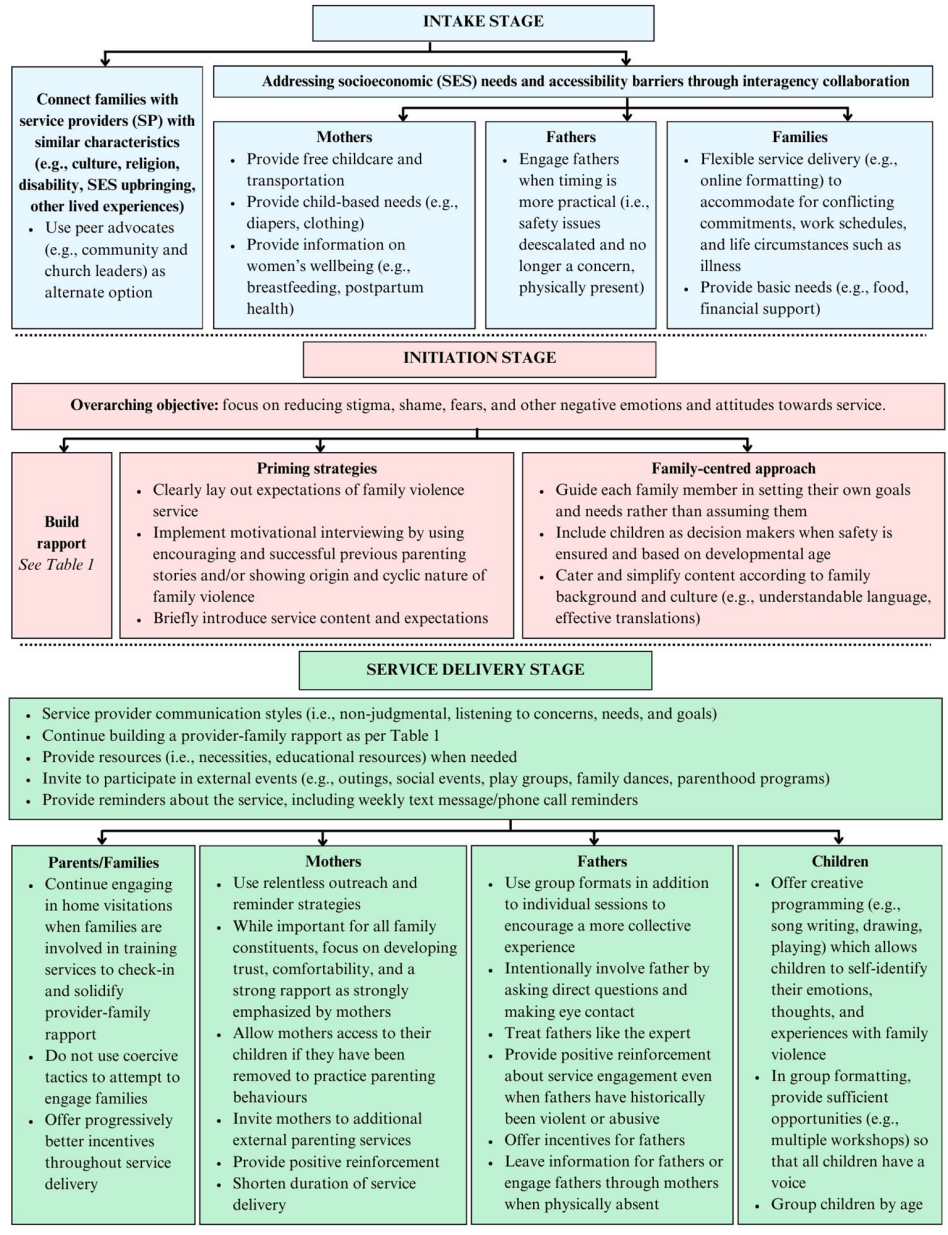
Flow diagram showing the recommended approach to engaging families in FV services, divided into intake, initiation, and service delivery stages.

Findings from this review may be arranged into three sections focused on the intake process, initiation of service, and service delivery, as summarized in **Figure 4**. Recommendations may be drawn to address each key touchpoint that may improve engagment in FV services.

#### Intake process

These findings suggest that providers with similar characteristics and lived experiences to the families they support are better positioned to draw from their own experience to create an empathetic and understanding environment for family engagement (67, 73). This aligns with existing literature demonstrating that representation among service providers enhances understanding between providers and families, particularly within healthcare and social service settings (48, 84). When providers with these characteristics are unavailable or too costly, FV services may consider integrating peer advocates with lived experience to help families feel understood while navigating services (73). By normalizing families’ experiences with FV through shared experience, engagement is more likely to occur.

Services that address and reduce socioeconomic inequities can improve family engagement in FV services by increasing accessibility (60, 63). Although socioeconomic challenges are well established as barriers to service engagement (85, 86), findings from this review demonstrated unique prioritizations for each group. For example, mothers emphasized the provision of childcare, transportation, and child-related basic needs (e.g., diapers) as facilitators to service engagement, while poverty, work schedules, and housing insecurity were described as barriers. Conversely, fathers emphasized food security as a facilitator to engagement, while employment demands served as a barrier. These findings reflect broader gendered roles in which mothers are expected to take care of their children while maintaining employment (87), whereas fathers are more frequently expected to act as financial providers (86). Accordingly, each parent’s socioeconomic needs must be addressed prior to commencing FV services, so that the focus is on service engagement rather than overcoming socioeconomic and accessibility challenges.

Online formats, identified by families generally and by fathers specifically as facilitators to engagement, may effectively reduce barriers associated with transportation, employment, and childcare. This is particularly relevant when sociocultural contexts, geography, and digital literacy are incorporated throughout implementation (88). Although no studies captured in this review identified online formats as a facilitator to mothers’ engagement specifically, several studies have elucidated reduced levels of child abuse among mothers enrolled in online parenting services (89), indicating that the accessibility of online formatting could be extended to mothers to promote engagement.

FV services may also consider providing financial supports, such as covering childcare or transportation costs, to facilitate engagement (90). These forms of support should also be viewed as upstream investments in prevention and early intervention by larger funding bodies such as governments and policymakers. Financially supporting families’ engagement in FV service can lead to reductions in FV occurrence and associated downstream costs (e.g., healthcare, legal, costs associated with loss of productivity).

For example, although focused specifically on IPV, Varcoe et al. (36) estimated that violence toward women resulted in an annual healthcare expenditure of CAD $13, 162.39 per woman, even under conservative assumptions. In this context, financial support received through FV services that meaningfully reduce exposure to violence may have downstream economic implications. To illustrate this potential, if a FV service cost CAD $10, 000.00 (hypothetical) annually per woman and was associated with the prevention of violence exposure, projected savings would approximate to CAD $3, 150 per woman per year. Given that there were approximately 78, 000 women who experienced IPV in 2011 in Canada (likely underestimated) (91), this illustrative calculation would correspond to approximately $245, 700, 000 CAD in potential avoided costs. These illustrative estimates are not intended as a formal cost-effective analysis or prediction of realized savings, but rather to highlight the potential magnitude of economic burden associated with FV under simplified assumptions.

Similarly, substantial cost reductions may be achieved by investing in prevention and early family support to reduce out-of-home child placements. As of 2025, Canadian foster caregivers in British Columbia received monthly maintenance payments of CAD $1, 549.20 for children aged 0–11 and $1, 726.33 for children aged 12-19 (92). A report from the Government of Canada indicate that at least 61, 104 children were receiving out-of-home care as of March 2022 (93), translating to approximately 1.1 billion Canadian dollars in funding allocated towards foster care services. Providing early support to families may reduce the need for out-of-home placement and thus lead to reduced governmental costs. Thus, supporting families experiencing FV not only promotes public health, but it also may yield significant, national economic gains.

#### Initiation of service

Studies emphasized the importance of provider-family rapport built on trust, consistent with broader literature (94). This also aligns with family-centred community care approaches, which emphasize creating and maintaining respectful relationships, adapting and contextualizing care, effective communication, and supporting familial autonomy (83). Trust is important as families often perceive child welfare services and other service providers as threatening because of the authority these systems can hold over family and child outcomes (94, 95). In contrast, coercive tactics such as threats, which undermined trust, were identified as barriers to engagement in FV services (58). Trust was facilitated by providers who were open, knowledgeable, reliable (i.e., following through with identified requests or plans), and culturally competent (61). When service providers lacked sufficient training to demonstrate openness and knowledge, participants were more likely to drop-out of services (64). Further, the importance of cultural competency in developing provider-family rapport aligns with literature demonstrating cultural differences in conceptualizing and understandings of FV, structures, and dynamics (96). Cultural competency involves the use of sensitive language and behaviours to address families’ preconceived notions of healthy family functioning without blaming, shaming, or attacking them (76). Main aspects of developing strong provider-family rapport are provided (**Table 1**).

**Table 1:** Important aspects to developing strong provider-family rapport.

Aspects of strong provider-family rapport
• Invite families to share their treatment goals • Make eye contact • Remember all family names • Remember each family member’s needs and objectives • Positive reinforcement through intentionally acknowledging, affirming, and encouraging parental effort and participation • Active listening • Following through with requests from parents • Share similar lived experiences when possible and/or draw from successful previous parenting stories • Self-disclosure • Be respectful to cultural differences and needs • Take time to show commitment and interest to families with warm, kind, and non-blaming attitudes • Build trust through provider self-disclosure • Ask direct and specific questions • For fathers, emphasizing the importance of fatherhood

Since priming approaches for mothers, fathers, and families were found to generally encourage engagement in FV services (59, 76, 97), whereas intense intake meetings and service initiation processes reduced engagement (61), it is recommended that service providers implement priming strategies (e.g., motivational interviewing, introductory sessions) prior to active involvement in services. Findings from related literature further support the importance of the first point of contact in optimizing the effectiveness and impacts of FV services (98). The use of priming strategies aligns with social cognitive theory (99), a theoretical framework that explains human behaviour as a function of one’s self-belief in overcoming challenges and expectations regarding outcomes (100). Moreover, social cognitive theory has been successfully applied to promote positive behavioural changes and outcomes across various contexts (101–105). While not yet explicitly evaluated in the context of FV, priming strategies appear to reflect key aspects of social cognitive theory by increasing parents’ knowledge and understanding of FV before encouraging behavioural change.

#### Service delivery

Mothers’ engagement in FV services was often hindered by fear of stigma or judgment (60). This finding aligns with existing literature focused on the “good mother” misconception (106). The “good mother” misconception is rooted in gendered constructs that uphold patriarchal expectations of motherhood, where unrealistically high parenting standards magnify mothers’ perceived mistakes (94, 106, 107). For example, mothers experiencing abuse have been blamed for being unable to protect their child (94). Moreover, mothers with mental health conditions are labelled as “crazy”, “unstable”, or “mad” (108), while those who pursue occupational roles are villainized (107). Mothers’ emphasis on favourable service provider characteristics and behaviours as prominent facilitators to FV service engagement therefore becomes understandable, as many mothers experience stigma and shame associated with societal pressures surrounding motherhood, which can undermine help-seeking behaviours (94). Ensuring that FV services address the “good mother” misconception throughout service delivery, while considering how these expectations may differ across cultures (109), may help to decrease mothers’ feelings of stigma and shame and facilitate engagement.

Relentless outreach, including ongoing follow-up attempts despite unsuccessful contact, was also identified as a facilitator to mothers’ engagement in FV services (75). This was particularly true for mothers experiencing compounding vulnerabilities (e.g., poverty) (75), paralleling other studies which show lower retention rates among individuals experiencing challenges such as financial insecurity (110, 111). Similarly, mothers whose children had been removed by child welfare services were more likely to engage in FV services when given opportunities to actively practice parenting skills with their children (76). Eliminating all contact with children reduces parents’ desire to engage in FV services (76). Therefore, mothers are more likely to engage when they are able to directly apply learned parenting skills through interactions with their children.

Experiences of stigma and shame were also evident among abusive fathers, who often found it difficult to discuss their FV experiences, goals, and behaviours, particularly within cultures where patriarchal values are deeply engrained within socioeconomical contexts (78, 79). While fathers identified positive provider characteristics as factors that reduced their internalized stigma and shame, fathers needed to be intentionally included throughout service content and delivery to feel important (72). This aligns with meta-synthesis findings suggesting that fathers are frequently overlooked within child welfare services (94), highlighting the need to reframe how services are delivered. This distinction is particularly important in comparison to mothers, who are often automatically viewed as the primary parent and therefore prioritized within child welfare services (112). Although mothers play a pertinent role in child development (e.g., breastfeeding, nurturing), greater paternal involvement in child-rearing has also been associated with more positive outcomes for both children (113, 114) and mothers (115). Therefore, FV services should be designed to support and include both mothers and fathers as equally important participants throughout service delivery.

Another factor that decreased fathers’ internalized stigma and shame and increased engagement in FV services was the use of group formats. Group settings reduced fathers’ feelings of isolation by shifting the experience from an individual to a collective one (60), particularly within cultures where FV is considered more taboo (79). Interviews conducted with fathers suggest that group programming can help to normalize, rather than pathologize, paternal anger, thereby reducing the stigma and shame that can prevent engagement (116). However, facilitators must be aware that group environments can be retraumatizing (117). When appropriately facilitated by a trained professional within supportive conditions, group formats may help fathers overcome internalized negative emotions and beliefs while promoting engagement in FV services.

### Family-centred, trauma-informed, and interagency approaches are foundational to all stages of FV services

Families, mothers, and fathers emphasized a family-centred community care approach as a facilitator to engagement which improves ongoing engagement in FV services (64), likely because it addresses families’ actual needs and concerns rather than perceived needs and concerns (94). For example, family-centered scheduling allows parents to participate together or separately based on work schedules, relationship status, and childcare responsibilities, thereby providing flexibility that supports ongoing retention in services (72). Moreover, a family centred-care approach places all family members, including children when appropriate, at the centre of decision-making, allowing families to identify desired outcomes and recognize when and how support is needed throughout the process (118). These principles directly reflect core components of family-centred care models, including adapting and contextualizing care and supporting family autonomy (83). Education and training for service providers should be strengthened through policies which clearly outline responsibilities related to implementing family-centred care. This includes mutually learning from family members, using language that promotes communication (119), empowering families through guidance in decision-making rather than assuming control, and expanding cultural awareness while recognizing when providers are limited (e.g., language barriers) in their capacity to engage with specific families (118).

McGinn et al. (120) conducted a systematic review examining outcomes for children at-risk of abuse or neglect involved in family group decision-making, revealing largely positive (e.g., reunification with families of origin), and, at times, negative implications (e.g., increased maltreatment) of doing so. However, McCafferty et al. (121) conducted an umbrella review examining children’s involvement in child welfare services and found that children’s voices are often not taken seriously and that relationships between children and providers are imperative for engagement. Together, these reviews and the present findings emphasize key factors that underlie a family-centred care approach. To fully implement family-centred care, providers must treat children as equal constituents throughout FV service decision-making and advocate for children when parents fail to acknowledge their children’s perspectives.

In some instances, families requiring multiple services to address complex needs (e.g. culture, disability, finances, gender, and substance use) may be better supported through interagency collaboration rather than cold referrals (71). Service providers identified collaboration across multiple agencies as important for effectively engaging and building working relationships with families experiencing FV (122). Given that families involved in the child welfare system are often referred to various services and agencies which creates confusion, hesitancy, and fear (80), maximizing community resources through interdisciplinary collaboration may better address the complex interplay of familial, personal, cultural, and psychological needs and enable more individualized care.

Moreover, a trauma-and-violence-informed care (TVIC) approach should underpin service provision within FV services. Although trauma-informed care focuses on reducing the risk of re-traumatization and fostering safe care environments, TVIC broadens this approach by also acknowledging the influence of ongoing structural and systematic inequities (123). TVIC emphasizes awareness of trauma and violence and their impacts, prioritizes physical, emotional, and cultural safety, promotes person-centered connection, and builds upon individual and family strengths (123). Further, services should employ TVIC through facilitating individualized care and referrals, rather than placing the burden on families to independently navigate fragmented systems of support (123). Davies et al. (124) identified several key principles underlying TVIC, including ensuring safety, emotional support, survivor flourishing, cultural safety and inclusivity, and accessible services. Ultimately, a TVIC aims to minimize the potential for re-traumatization and improve access to supportive, equitable, and responsive care for trauma survivors (124). As such, TVIC principles should be embedded across FV service delivery, provider training, and engagement strategies.

### Future research: implementation science and evaluation

Most of the included studies primarily focused on describing service outcomes rather than evaluating strategies specifically designed to improve engagement in FV services (68). Therefore, future research should test engagement strategies, such as those suggested in **Figure 4**, to support improved participation and retention in FV services. In particular, testing strategies among groups experiencing differing vulnerabilities would be valuable (125). Co-designing interventions with service providers and families experiencing FV may further improve long-term uptake and sustainability of engagement strategies. In addition, future research should move beyond identifying engagement barriers by examining the contextual barriers and facilitators that influence FV service delivery to better understand how evidence-informed strategies can be feasibly implemented, sustained, and adapted across diverse FV service contexts and settings. Moreover, there remains a gap in research evaluating children’s perspectives within FV services. Including children in qualitative research and gathering their direct perspectives is essential for advancing understanding of factors that facilitate successful family engagement in relevant services. Employing implementation science methods to identify contextual facilitators, barriers, and key outcomes may further support the development of evidence-informed solutions to better care for families experiencing FV (126–128).

### Limitations and strengths

Although it is possible that some relevant studies were not captured, the search strategy was developed with guidance from an expert health sciences librarian (KAH) and conducted systematically across multiple databases to enhance the rigor and comprehensiveness of the search process. Most studies captured in this review evaluated engagement strategies in the United States. Because cultural differences, healthcare infrastructure, service accessibility, and perceptions of family violence vary across settings, these strategies may not be transferable to low- and middle-income countries or other sociocultural contexts. Nevertheless, factors such as trust and family-centred approaches were consistently identified across studies, suggesting that they may serve as foundational principles when adapting strategies to different countries and contexts. Moreover, most studies did not address the severity of violence targeted by services. As a result, it remains unclear whether distinct facilitators or barriers exist for families experiencing higher versus lower levels of violence, and how these differences may influence family engagement in relevant services. Lastly, findings are likely not generalizable to certain populations (e.g., sexual and gender minority parents) as these groups experience unique challenges and forms of abuse that were not adequately considered within included studies.

## Conclusion

This realist review, conducted systematically, synthesized literature on barriers and facilitators that influence families’ engagement in FV services, guiding the recommendation of a detailed strategy focused on intake (e.g., addressing parent-specific socioeconomic concerns, connecting families with provider who share similar identities or lived experiences), initiation (e.g., priming strategies, family-centred care approaches), and service delivery (e.g., relentless outreach strategies, group formats for fathers, creative programming for children). Implementation science methods are recommended to evaluate these proposed strategies and further identify evidence-based approaches to promote effective engagement of families, mothers, fathers, and children in FV services.

## Data Availability

The original contributions presented in the study are included in the article/**Supplementary Material**. Further inquiries can be directed to the corresponding author.
