# Supplementary material for: Increasing familial engagement in family violence services: a systematically conducted realist review of barriers and facilitators
Source: Front Psychiatry. 2026 Jul 8;17:1588381. doi: 10.3389/fpsyt.2026.1588381 (PMC13388260; doi:10.3389/fpsyt.2026.1588381)
Supplement: Supplementary file 1 [file Table1.docx]

**Table A.**

Search strategy for APA PsycInfo (OVID)

| **#** | **Searches** |
| --- | --- |
| 1 | domestic violence/ or intimate partner violence/ |
| 2 | child abuse/ or child neglect/ |
| 3 | physical abuse/ or emotional abuse/ or verbal abuse/ or punishment/ |
| 4 | exp sexual abuse/ |
| 5 | (child* adj2 (abus* or maltreat* or neglect*)).tw,id. |
| 6 | ((intimate or partner or spouse or spousal or family or familial or domestic or interpersonal or gender or inter parental or interparental) adj2 (abus* or violence)).tw,id. |
| 7 | ((sex* or physical or psychological or verbal or emotional) adj2 (abus* or violence)).tw,id. |
| 8 | ((physical or emotional or psychological) adj2 neglect*).tw,id. |
| 9 | ((corporal or physical) adj2 punishment).tw,id. |
| 10 | ((verbal or physical or psychological) adj2 aggression).tw,id. |
| 11 | or/1-10 |
| 12 | family/ or biological family/ or dysfunctional family/ or family members/ or family relations/ or nuclear family/ or stepfamily/ |
| 13 | father child relations/ or parent child relations/ or mother child relations/ |
| 14 | fathers/ or parents/ or adolescent fathers/ or single fathers/ |
| 15 | mothers/ or adolescent mothers/ or single mothers/ or unwed mothers/ |
| 16 | (family or families or father* or mother*).tw,id. |
| 17 | (family or families or father* or stepfather* or step-father* or mother* or stepmother* or step-mother*).tw,id. |
| 18 | or/12-17 |
| 19 | exp intervention/ |
| 20 | program development/ or educational program planning/ or exp educational programs/ or exp mental health programs/ or exp program evaluation/ or exp social programs/ |
| 21 | health care services/ or behavioral health services/ or mental health services/ or community services/ or outreach programs/ or social services/ |
| 22 | (intervention* or program* or education* or service* or strateg*).tw,id. |
| 23 | or/19-22 |
| 24 | psychological engagement/ |
| 25 | motivation/ or collaboration/ |
| 26 | (engag* or participat* or involv* or recruit* or motivat* or collaborat*).ti,id. |
| 27 | or/24-26 |
| 28 | 11 and 18 and 23 and 27 |
| 29 | greenbook.tw,id. |
| 30 | 28 or 29 |
| 31 | limit 30 to peer reviewed journal |

**Table B.**

Search strategy for CINAHL Plus with Full Text (Ebsco)
**Search modes** - Find all my search terms

| \| **#** \| **Query** \| \| --- \| --- \| \| S32 \| S29 OR S30 Limiters - Scholarly (Peer Reviewed) Journals \| \| S31 \| S29 OR S30 \| \| S30 \| TI greenbook OR AB greenbook \| \| S29 \| S12 AND S20 AND S25 AND S28 \| \| S28 \| S26 OR S27 \| \| S27 \| TI ( (engag* or participat* or involv* or recruit* or motivat* or collaborat*) ) \| \| S26 \| (MH "Collaboration") \| \| S25 \| S21 OR S22 OR S23 OR S24 \| \| S24 \| TI ( (intervention* or program* or education* or service* or strateg*) ) AND AB ( (intervention* or program* or education* or service* or strateg*) ) \| \| S23 \| (MH "Community Mental Health Services") OR (MH "Correctional Health Services") OR (MH "Health Services Accessibility") OR (MH "Community Health Services") OR (MH "Mental Health Services") OR (MH "Child Health Services") OR (MH "Family Services") \| \| S22 \| (MH "Program Development") OR (MH "Program Planning") OR (MH "Community Programs") OR (MH "Mental Health") OR (MH "Substance Use Rehabilitation Programs") OR (MH "Drug Rehabilitation Programs") \| \| S21 \| (MH "Crisis Intervention") OR (MH "Psychosocial Intervention") OR (MH "Intervention Scheme (Omaha)") OR (MH "Early Intervention") OR (MH "Early Childhood Intervention") \| \| S20 \| S13 OR S14 OR S15 OR S16 OR S17 OR S18 OR S19 \| \| S19 \| TI ( (family or families or father* or stepfather* or step-father* or mother* or stepmother* or step-mother*) ) OR AB ( (family or families or father* or stepfather* or step-father* or mother* or stepmother* or step-mother*) ) \| \| S18 \| TI ( (family or families or father* or mother*) ) OR AB ( (family or families or father* or mother*) ) \| \| S17 \| (MH "Adolescent Mothers") OR (MH "Mothers") \| \| S16 \| (MH "Parents") OR (MH "Biological Parents") OR (MH "Stepfamilies") \| \| S15 \| (MH "Fathers") OR (MH "Single Parent") OR (MH "Adolescent Fathers") \| \| S14 \| (MH "Parent-Child Relations") OR (MH "Mother-Child Relations") OR (MH "Father-Child Relations") \| \| S13 \| (MH "Family") OR (MH "Nuclear Family") OR (MH "Family Relations") OR (MH "Dysfunctional Family") \| \| S12 \| S1 OR S2 OR S3 OR S4 OR S5 OR S6 OR S7 OR S8 OR S9 OR S10 OR S11 \| \| S11 \| TI ( ((verbal or physical or psychological) N2 aggression) ) OR AB ( ((verbal or physical or psychological) N2 aggression) ) \| \| S10 \| TI ( ((corporal or physical) N2 punishment) ) OR AB ( ((corporal or physical) N2 punishment) ) \| \| S9 \| TI ( ((physical or emotional or psychological) N2 neglect*) ) OR AB ( ((physical or emotional or psychological) N2 neglect*) ) \| \| S8 \| TI ( ((sex* or physical or psychological or verbal or emotional) N2 (abus* or violence)) ) OR AB ( ((sex* or physical or psychological or verbal or emotional) N2 (abus* or violence)) ) \| \| S7 \| TI ( ((intimate or partner or spouse or spousal or family or familial or domestic or interpersonal or gender or inter parental or interparental) N2 (abus* or violence)) ) OR AB ( ((intimate or partner or spouse or spousal or family or familial or domestic or interpersonal or gender or inter parental or interparental) N2 (abus* or violence)) ) \| \| S6 \| TI ( (child* N2 (abus* or maltreat* or neglect*)) ) OR AB ( (child* N2 (abus* or maltreat* or neglect*)) ) \| \| S5 \| (MH "Sexual Abuse+") \| \| S4 \| (MH "Punishment") \| \| S3 \| (MH "Emotional Abuse") OR (MH "Verbal Abuse") \| \| S2 \| (MH "Child Abuse") OR (MH "Neglect (Omaha)") \| \| S1 \| (MH "Domestic Violence") OR (MH "Intimate Partner Violence") \| |
| --- | --- | --- | --- | --- | --- | --- | --- | --- | --- | --- | --- | --- | --- | --- | --- | --- | --- | --- | --- | --- | --- | --- | --- | --- | --- | --- | --- | --- | --- | --- | --- | --- | --- | --- | --- | --- | --- | --- | --- | --- | --- | --- | --- | --- | --- | --- | --- | --- | --- | --- | --- | --- | --- | --- | --- | --- | --- | --- | --- | --- | --- | --- | --- | --- | --- | --- |

**Table C.**

Search strategy for Social Work Abstracts (Ebsco)
**Search modes** - Find all my search terms

| \|  \| **Query** \| \| --- \| --- \| \| S29 \| S26 OR S27 Limiters - Scholarly (Peer Reviewed) Journals \| \| S28 \| S26 OR S27 \| \| S27 \| TI greenbook OR AB greenbook OR greenbook \| \| S26 \| S11 AND S17 AND S22 AND S25 \| \| S25 \| S23 OR S24 \| \| S24 \| TI engag* or participat* or involv* or recruit* or motivat* or collaborat* \| \| S23 \| TI psychological engagement OR AB psychological engagement OR KW psychological engagement \| \| S22 \| S18 OR S19 OR S20 OR S21 \| \| S21 \| TI ( intervention* or program* or education* or service* or strateg* ) OR AB ( intervention* or program* or education* or service* or strateg* ) OR KW ( intervention* or program* or education* or service* or strateg* ) \| \| S20 \| TI ( health care services or behavioral health services or mental health services or community services or outreach programs or social services ) OR AB ( health care services or behavioral health services or mental health services or community services or outreach programs or social services ) OR KW ( health care services or behavioral health services or mental health services or community services or outreach programs or social services ) \| \| S19 \| TI ( program development or educational program planning or educational programs or mental health programs or program evaluation or social programs ) OR AB ( program development or educational program planning or educational programs or mental health programs or program evaluation or social programs ) OR KW ( program development or educational program planning or educational programs or mental health programs or program evaluation or social programs ) \| \| S18 \| TI intervention OR AB intervention OR KW intervention \| \| S17 \| S12 OR S13 OR S14 OR S15 OR S16 \| \| S16 \| TI ( stepfather* or step-father* or mother* or stepmother* or step-mother* or father* or families ) OR AB ( stepfather* or step-father* or mother* or stepmother* or step-mother* or father* or families ) OR KW ( stepfather* or step-father* or mother* or stepmother* or step-mother* or father* or families ) \| \| S15 \| TI ( mothers or adolescent mothers or single mothers or unwed mothers ) OR AB ( mothers or adolescent mothers or single mothers or unwed mothers ) OR KW ( mothers or adolescent mothers or single mothers or unwed mothers ) \| \| S14 \| TI ( fathers or parents or adolescent fathers or single fathers ) OR AB ( fathers or parents or adolescent fathers or single fathers ) OR KW ( fathers or parents or adolescent fathers or single fathers ) \| \| S13 \| TI ( father child relations or parent child relations or mother child relations ) OR AB ( father child relations or parent child relations or mother child relations ) OR KW ( father child relations or parent child relations or mother child relations ) \| \| S12 \| TI ( family or biological family or dysfunctional family or family members or family relations or nuclear family or stepfamily ) OR AB ( family or biological family or dysfunctional family or family members or family relations or nuclear family or stepfamily ) OR KW ( family or biological family or dysfunctional family or family members or family relations or nuclear family or stepfamily ) \| \| S11 \| S1 OR S2 OR S3 OR S4 OR S5 OR S6 OR S7 OR S8 OR S9 OR S10 \| \| S10 \| TI ( ((physical or emotional or psychological) N2 neglect*) ) OR AB ( ((physical or emotional or psychological) N2 neglect*) ) OR KW ( ((physical or emotional or psychological) N2 neglect*) ) \| \| S9 \| TI ( ((verbal or physical or psychological) N2 aggression) ) OR AB ( ((verbal or physical or psychological) N2 aggression) ) OR KW ( ((verbal or physical or psychological) N2 aggression) ) \| \| S8 \| TI ( ((corporal or physical) N2 punishment) ) OR AB ( ((corporal or physical) N2 punishment) ) OR KW ( ((corporal or physical) N2 punishment) ) \| \| S7 \| TI ( ((sex* or physical or psychological or verbal or emotional) N2 (abus* or violence)) ) OR AB ( ((sex* or physical or psychological or verbal or emotional) N2 (abus* or violence)) ) OR KW ( ((sex* or physical or psychological or verbal or emotional) N2 (abus* or violence)) ) \| \| S6 \| TI ( ((intimate or partner or spouse or spousal or family or familial or domestic or interpersonal or gender or inter parental or interparental) N2 (abus* or violence)) ) OR AB ( ((intimate or partner or spouse or spousal or family or familial or domestic or interpersonal or gender or inter parental or interparental) N2 (abus* or violence)) ) OR KW ( ((intimate or partner or spouse or spousal or family or familial or domestic or interpersonal or gender or inter parental or interparental) N2 (abus* or violence)) ) \| \| S5 \| TI ( (child* N2 (abus* or maltreat* or neglect*)) ) OR AB ( (child* N2 (abus* or maltreat* or neglect*)) ) OR KW ( (child* N2 (abus* or maltreat* or neglect*)) ) \| \| S4 \| TI sexual abuse OR AB sexual abuse OR KW sexual abuse \| \| S3 \| TI ( physical abuse or emotional abuse or verbal abuse or punishment ) OR AB ( physical abuse or emotional abuse or verbal abuse or punishment ) OR KW ( physical abuse or emotional abuse or verbal abuse or punishment ) \| \| S2 \| TI ( child abuse or child neglect ) OR AB ( child abuse or child neglect ) OR KW ( child abuse or child neglect ) \| \| S1 \| TI ( domestic violence or intimate partner violence ) OR AB ( domestic violence or intimate partner violence ) OR KW ( domestic violence or intimate partner violence ) \| |
| --- | --- | --- | --- | --- | --- | --- | --- | --- | --- | --- | --- | --- | --- | --- | --- | --- | --- | --- | --- | --- | --- | --- | --- | --- | --- | --- | --- | --- | --- | --- | --- | --- | --- | --- | --- | --- | --- | --- | --- | --- | --- | --- | --- | --- | --- | --- | --- | --- | --- | --- | --- | --- | --- | --- | --- | --- | --- | --- | --- | --- |

**Table D.**

Search strategy for Family and Society Studies Worldwide (Ebsco)
**Search modes** - Find all my search terms

| \| **#** \| **Query** \| \| --- \| --- \| \| S29 \| S26 OR S27 Limiters - Scholarly (Peer Reviewed) Journals \| \| S28 \| S26 OR S27 \| \| S27 \| TI greenbook OR AB greenbook OR KW greenbook \| \| S26 \| S11 AND S17 AND S22 AND S25 \| \| S25 \| S23 OR S24 \| \| S24 \| TI engag* or participat* or involv* or recruit* or motivat* or collaborat* \| \| S23 \| TI psychological engagement OR AB psychological engagement OR KW psychological engagement \| \| S22 \| S18 OR S19 OR S20 OR S21 \| \| S21 \| TI ( intervention* or program* or education* or service* or strateg* ) OR AB ( intervention* or program* or education* or service* or strateg* ) OR KW ( intervention* or program* or education* or service* or strateg* ) \| \| S20 \| TI ( health care services or behavioral health services or mental health services or community services or outreach programs or social services ) OR AB ( health care services or behavioral health services or mental health services or community services or outreach programs or social services ) OR KW ( health care services or behavioral health services or mental health services or community services or outreach programs or social services ) \| \| S19 \| TI ( program development or educational program planning or educational programs or mental health programs or program evaluation or social programs ) OR AB ( program development or educational program planning or educational programs or mental health programs or program evaluation or social programs ) OR KW ( program development or educational program planning or educational programs or mental health programs or program evaluation or social programs ) \| \| S18 \| TI intervention OR AB intervention OR KW intervention \| \| S17 \| S12 OR S13 OR S14 OR S15 OR S16 \| \| S16 \| TI ( stepfather* or step-father* or mother* or stepmother* or step-mother* or father* or families ) OR AB ( stepfather* or step-father* or mother* or stepmother* or step-mother* or father* or families ) OR KW ( stepfather* or step-father* or mother* or stepmother* or step-mother* or father* or families ) \| \| S15 \| TI ( mothers or adolescent mothers or single mothers or unwed mothers ) OR AB ( mothers or adolescent mothers or single mothers or unwed mothers ) OR KW ( mothers or adolescent mothers or single mothers or unwed mothers ) \| \| S14 \| TI ( fathers or parents or adolescent fathers or single fathers ) OR AB ( fathers or parents or adolescent fathers or single fathers ) OR KW ( fathers or parents or adolescent fathers or single fathers ) \| \| S13 \| TI ( father child relations or parent child relations or mother child relations ) OR AB ( father child relations or parent child relations or mother child relations ) OR KW ( father child relations or parent child relations or mother child relations ) \| \| S12 \| TI ( family or biological family or dysfunctional family or family members or family relations or nuclear family or stepfamily ) OR AB ( family or biological family or dysfunctional family or family members or family relations or nuclear family or stepfamily ) OR KW ( family or biological family or dysfunctional family or family members or family relations or nuclear family or stepfamily ) \| \| S11 \| S1 OR S2 OR S3 OR S4 OR S5 OR S6 OR S7 OR S8 OR S9 OR S10 \| \| S10 \| TI ( ((physical or emotional or psychological) N2 neglect*) ) OR AB ( ((physical or emotional or psychological) N2 neglect*) ) OR KW ( ((physical or emotional or psychological) N2 neglect*) ) \| \| S9 \| TI ( ((verbal or physical or psychological) N2 aggression) ) OR AB ( ((verbal or physical or psychological) N2 aggression) ) OR KW ( ((verbal or physical or psychological) N2 aggression) ) \| \| S8 \| TI ( ((corporal or physical) N2 punishment) ) OR AB ( ((corporal or physical) N2 punishment) ) OR KW ( ((corporal or physical) N2 punishment) ) \| \| S7 \| TI ( ((sex* or physical or psychological or verbal or emotional) N2 (abus* or violence)) ) OR AB ( ((sex* or physical or psychological or verbal or emotional) N2 (abus* or violence)) ) OR KW ( ((sex* or physical or psychological or verbal or emotional) N2 (abus* or violence)) ) \| \| S6 \| TI ( ((intimate or partner or spouse or spousal or family or familial or domestic or interpersonal or gender or inter parental or interparental) N2 (abus* or violence)) ) OR AB ( ((intimate or partner or spouse or spousal or family or familial or domestic or interpersonal or gender or inter parental or interparental) N2 (abus* or violence)) ) OR KW ( ((intimate or partner or spouse or spousal or family or familial or domestic or interpersonal or gender or inter parental or interparental) N2 (abus* or violence)) ) \| \| S5 \| TI ( (child* N2 (abus* or maltreat* or neglect*)) ) OR AB ( (child* N2 (abus* or maltreat* or neglect*)) ) OR KW ( (child* N2 (abus* or maltreat* or neglect*)) ) \| \| S4 \| TI sexual abuse OR AB sexual abuse OR KW sexual abuse \| \| S3 \| TI ( physical abuse or emotional abuse or verbal abuse or punishment ) OR AB ( physical abuse or emotional abuse or verbal abuse or punishment ) OR KW ( physical abuse or emotional abuse or verbal abuse or punishment ) \| \| S2 \| TI ( child abuse or child neglect ) OR AB ( child abuse or child neglect ) OR KW ( child abuse or child neglect ) \| \| S1 \| TI ( domestic violence or intimate partner violence ) OR AB ( domestic violence or intimate partner violence ) OR KW ( domestic violence or intimate partner violence ) \| |
| --- | --- | --- | --- | --- | --- | --- | --- | --- | --- | --- | --- | --- | --- | --- | --- | --- | --- | --- | --- | --- | --- | --- | --- | --- | --- | --- | --- | --- | --- | --- | --- | --- | --- | --- | --- | --- | --- | --- | --- | --- | --- | --- | --- | --- | --- | --- | --- | --- | --- | --- | --- | --- | --- | --- | --- | --- | --- | --- | --- | --- |

**Table E.**

Search strategy for SocINDEX with Full Text (Ebsco)
**Search modes** - Find all my search terms

| \| **#** \| **Query** \| \| --- \| --- \| \| S30 \| S27 OR S28 Limiters - Scholarly (Peer Reviewed) Journals \| \| S29 \| S27 OR S28 \| \| S28 \| TI greenbook OR AB greenbook OR KW greenbook \| \| S27 \| S11 AND S18 AND S23 AND S26 \| \| S26 \| S24 OR S25 \| \| S25 \| TI (engag* or participat* or involv* or recruit* or motivat* or collaborat*) \| \| S24 \| DE "MOTIVATION (Psychology)" \| \| S23 \| S19 OR S20 OR S21 OR S22 \| \| S22 \| TI ( (intervention* or program* or education* or service* or strateg*) ) OR AB ( (intervention* or program* or education* or service* or strateg*) ) OR KW ( (intervention* or program* or education* or service* or strateg*) ) \| \| S21 \| DE "MENTAL health services" OR DE "COMMUNITY mental health services" OR DE "PREVENTIVE health services" \| \| S20 \| DE "COMMUNITY services" OR DE "OUTREACH programs" OR DE "CHILD services" OR DE "COMMUNITY health services" \| \| S19 \| DE "INTERVENTION (Social services)" OR DE "HEALTH care intervention (Social services)" OR DE "HEALTH care intervention (Social services)" \| \| S18 \| S12 OR S13 OR S14 OR S15 OR S16 OR S17 \| \| S17 \| TI ( (family or families or father* or stepfather* or step-father* or mother* or stepmother* or step-mother*) ) OR AB ( (family or families or father* or stepfather* or step-father* or mother* or stepmother* or step-mother*) ) OR KW ( (family or families or father* or stepfather* or step-father* or mother* or stepmother* or step-mother*) ) \| \| S16 \| TI ( (family or families or father* or mother*) ) OR AB ( (family or families or father* or mother*) ) OR KW ( (family or families or father* or mother*) ) \| \| S15 \| DE "MOTHERS" OR DE "SINGLE mothers" OR DE "STAY-at-home mothers" OR DE "UNMARRIED mothers" \| \| S14 \| DE "FATHERS" OR DE "SINGLE fathers" OR DE "STAY-at-home fathers" OR DE "FATHER-child relationship" \| \| S13 \| DE "PARENT-child relationships" OR DE "MOTHER-child relationship" OR DE "PARENT-infant relationships" OR DE "STEPFATHERS" OR DE "STEPMOTHERS" OR DE "FAMILY conflict" OR DE "FAMILY relations" \| \| S12 \| DE "EXTENDED families" OR DE "STEPFAMILIES" OR DE "FAMILIES" OR DE "SUPPORT (Domestic relations)" OR DE "FAMILY roles" OR DE "FAMILY conflict" \| \| S11 \| S1 OR S2 OR S3 OR S4 OR S5 OR S6 OR S7 OR S8 OR S9 OR S10 \| \| S10 \| TI ( ((verbal or physical or psychological) N2 aggression) ) OR AB ( ((verbal or physical or psychological) N2 aggression) ) OR KW ( ((verbal or physical or psychological) N2 aggression) ) \| \| S9 \| TI ( ((corporal or physical) N2 punishment) ) OR AB ( ((corporal or physical) N2 punishment) ) OR KW ( ((corporal or physical) N2 punishment) ) \| \| S8 \| TI ( ((physical or emotional or psychological) N2 neglect*) ) OR AB ( ((physical or emotional or psychological) N2 neglect*) ) OR KW ( ((physical or emotional or psychological) N2 neglect*) ) \| \| S7 \| TI ( (sex* or physical or psychological or verbal or emotional) N2 (abus* or violence)) ) OR AB ( (sex* or physical or psychological or verbal or emotional) N2 (abus* or violence)) ) OR KW ( (sex* or physical or psychological or verbal or emotional) N2 (abus* or violence)) ) \| \| S6 \| TI ( ((intimate or partner or spouse or spousal or family or familial or domestic or interpersonal or gender or inter parental or interparental) N2 (abus* or violence)) ) OR AB ( ((intimate or partner or spouse or spousal or family or familial or domestic or interpersonal or gender or inter parental or interparental) N2 (abus* or violence)) ) OR KW ( ((intimate or partner or spouse or spousal or family or familial or domestic or interpersonal or gender or inter parental or interparental) N2 (abus* or violence)) ) \| \| S5 \| TI ( (child* N2 (abus* or maltreat* or neglect*)) ) OR AB ( (child* N2 (abus* or maltreat* or neglect*)) ) OR KW ( (child* N2 (abus* or maltreat* or neglect*)) ) \| \| S4 \| DE "ADULT child sexual abuse victims" OR DE "SOCIAL work with adult child sexual abuse victims" OR DE "SPOUSES of adult child sexual abuse victims" OR DE "CHILD sexual abuse" OR DE "SEXUAL abuse victims" OR DE "SEXUALLY abused children" OR DE "PARENTS of sexually abused children" OR DE "SEXUALLY abused boys" OR DE "SEXUALLY abused girls" \| \| S3 \| (DE "EMOTIONAL deprivation" OR DE "PSYCHOLOGICAL abuse" OR DE "PHYSICAL abuse") OR (DE "CORPORAL punishment") \| \| S2 \| DE "CHILD abuse" OR DE "ABUSED children" OR DE "CHILD sexual abuse" OR DE "PSYCHOLOGICAL child abuse" \| \| S1 \| DE "INTIMATE partner violence" OR DE "DOMESTIC violence" \| |
| --- | --- | --- | --- | --- | --- | --- | --- | --- | --- | --- | --- | --- | --- | --- | --- | --- | --- | --- | --- | --- | --- | --- | --- | --- | --- | --- | --- | --- | --- | --- | --- | --- | --- | --- | --- | --- | --- | --- | --- | --- | --- | --- | --- | --- | --- | --- | --- | --- | --- | --- | --- | --- | --- | --- | --- | --- | --- | --- | --- | --- | --- | --- |
