# Supplementary material for: Increasing familial engagement in family violence services: a systematically conducted realist review of barriers and facilitators
Source: Front Psychiatry. 2026 Jul 8;17:1588381. doi: 10.3389/fpsyt.2026.1588381 (PMC13388260; doi:10.3389/fpsyt.2026.1588381)
Supplement: Supplementary file 2 [file Table2.docx]

**Table 1.** Information on included studies (n=24).

| **Author, Date of Publication, and Country** | **Study Design** | **Sample**  **Size** | **Engagement Strategy Under Study** | **Relevant Outcome Measures** | **Barriers and Facilitators to Family Violence Engagement** |
| --- | --- | --- | --- | --- | --- |
| **Services Targeting Parents and/or Families (n=5)** | | | | | |
| *Balgopal et al., 1989, United States* | Quasi-Experimental; one group pretest-posttest | 34 abusive and neglective parents | -Parenting skills training program (2 hours/week x 8 weeks) and regularly scheduled home visits  -A contract was signed to ensure parents followed certain criteria (e.g., attending at least six of the eight training sessions, being at home when home visitations were planned)  -Caseworkers groomed child welfare services to acknowledge that resistance would be faced by parents | Attendance, engagement in training, attitudes towards home visits, and training | -High attendance rates (participants attended 83% of sessions)  - Low dropout rate (6% of participants)  -Home visits predisposed parents to be more open to learning during the training sessions  - Intial concerns about home visits being applicable to parents and about having a provider come to their home due to privacy concerns  - After program, parents valued home visits as it was personalized, they felt truly valued, and felt more comfortable  -Overall, increased engagement in the treatment program |
| *Chaffin et al., 2009, United States* | Crossover randomized controlled trial | 192 parents involved with child welfare services | -Targets parents of children referred for neglect and/or physical abuse  -Testing a motivational orientation intervention against standard orientation and an adjunct Parent–Child Interaction Therapy (PCIT) or standard didactic parent training group | Parenting program drop-out over time, changes in readiness for change over time, and barriers to engagement | -Combination of a motivational orientation with the PCIT group produced increased retention rates among individuals with low-to-medium motivation initially (85% vs. 61%)  -Improvement was not due to the motivational orientation or PCIT group alone, but rather a synergy between the two |
| *Damashek et al., 2012, United States* | Randomized controlled trial | 1305 parents involved with child welfare services | -Focused on provider-caregiver relationship (for caregivers in child welfare services)  -The effect of service type (manualized vs. non-manualized) perceived provider cultural competence on client satisfaction and engagement with services | Client perspective of provider cultural competence, client goal attainment, and client satisfaction | -Higher provider cultural competence was associated with increased goal attainment and client satisfaction  -Increased goal attainment was found in those that received the manualized treatment |
| *Fitz-Symonds et al., 2024, United Kingdom* | Realist methodology; interviews and focus groups | 12 parents;  12 peer parent advocates | **-**Peer Parental Advocacy (PPA) programmes  -PPA acts as bridge between parents and professionals by providing emotional and informational support to parents | Parental engagement in program, mechanisms for engagement through focus groups with peer advocates and professionals | -Peer advocate has lived experience to help empathise with parents  -Three intervention stages to improve engagement  (1) Pre-advocate involvement –PPA training, preparing parents for involvement (2) During advocate involvement – PPA provides emotional and informational support to parent in CPS (3) Ongoing support – PPA continues to support parents  -Identified mechanisms for PPA implementation to enhance parental engagement: (1) Building and facilitating trust -reducing power imbalance, stigma, and emotional difficulties. PPA’s were able to build trust to engage parents (2) Active PPA engagement with parents – initial pre-involvement phase is key to prepare parent for active engagement. Emotional and informational support by PPA improves communication. Reduces complexity of the system, complex terminology for parents, allowing meetings to feel more accessible (3) Enabling effective communication and participation – ensuring parents’ voices are heard by allowing parents to express feelings and wishes to PPA (4) Effectively supporting advocates to do good work – empowering PPAs through supervision |
| *Strolin-Goltzman et al., 2023, United States* | Program evaluation | 40 parents | -New parenting curriculum targeting parents in the child welfare system  -Breakthrough Parent Curriculum: Navigating Trauma Across Generations  -Curriculum aimed at strengthening family connection and addressing trauma in parent and child | Parent satisfaction and perceptions, child and parent outcomes | -High completion rate (72%) based on attending seven of 10 sessions  -High levels of satisfaction (mean at or above 4.7 on 5-point scale)  -Flexibility and accessibility of online format were identified as helpful by participants  -Participants suggested more classes to be available  -Parents reported significant increase in knowledge and skills related to parenting and trauma  -Parents felt more connected to children after treatment  -Parent facilitator had lived experienced, improved engagement among participants  -Online formatting improved accessibility while allowing parents to connect with others |
| **Services Targeting Mothers (n=7)** | | | | | |
| *Chablani and Spinney, 2011, United States* | Program evaluation | 81 mothers | -Circle of Care project targets high-risk young parents including those who have been victims of abuse and neglect  -Circle of Care Project has intensive strategy for engaging high-risk parents | Two engagement goals: (1) engagement into programming (2) continued engagement | -Participant retention rate high (90%) after one year  -Circle of Care Project recruited and enrolled 81 young mothers into program, exceeding the anticipated number of participants.  -Engagement strategies: (1) relentless outreach to continue reconnecting with mothers throughout the program (2) building transformational relationships with workers by building trust and leveraging this to promote behavioral changes (3) stage-based programming to match parents into an appropriate program stage for individualized care |
| *Girvin et al., 2007, United States* | Randomized controlled trial | 136 parents (98% mothers) | -Family connections is a social work child neglect preventive intervention | Factors that predict program completion among families, client satisfaction of worker-client relationship, service completion | -Increased service completion rates among those with higher positive perceptions of caregiver-provider relationship, greater depressive symptoms at intake, and shorter service linked predicter greater participation in Family Connections |
| *Farrell et al., 2012, United States* | Mixed-method | 41 parents (40 mothers) involved with child welfare services | -Targets parents with parenting, substance use, and/or mental health challenges  -Client perspective on the level and nature (quality) of their engagement | Parent Engagement Measure to create mean engagement score and interview of 7 main themes | -Low engagement was associated with poorer relationships with caseworkers and/or unclear caseworker roles  -Higher engagement scores were associated with positive relationships and confidence in their caseworker, clear understanding of the caseworker's role, and tangible outcomes |
| *Fogarty et al., 2023, Australia* | Qualitative; Semi-structured interviews | 7 mothers | -Targets families with children between 0-4 years of age who have concerns of child maltreatment  -The Home Parenting Education Support program (HoPES) aims to improve: (1) parenting knowledge, independence, and skills, (2) parenting practices, and (3) parent-child interactions | Facilitators and barriers to engagement in HoPES | -The following were described as facilitators to engagement: (1) clinician behaviours (normalizing parenting challenges, non-judgmental responses, knowledgeable, and reliability); (2) treatment relevance and acceptability (focusing on play, tailoring the program to meet family needs, using materials, using a strengths-based approach, and focusing on skill development); (3) strong therapeutic relationship (positive relationships); (4) parent cognitions and beliefs about treatment (parental perceptions regarding the need for support, their openness and motivation, and committing to the program); (5) program delivery (flexibility, home-visiting, informative intake meetings, clear program expectations, and effective referral process)  -Barriers to engagement included life stress, child illness, competing demands and appointments, difficulty engaging partners, lack of coverage on different parental issues, intensity of intake meeting, fear of being judged, lack of trust, lack of awareness, inflexible program cycles, poor referral process, and overall program duration |
| *Leckey et al., 2021, Ireland* | Qualitative; semi-structured interviews and questionnaires | 12 mothers | -Children At Risk Model (ChARM) model includes four core components: (1) a Positive Life Skills Program (PLSP) 4 weeks (2) Incredible Years Parenting Program (IYPP) 14 weeks (3) home visits (4) referrals to community services  -PLSP is a parent-engagement program designed for parents who have barriers to engagement | Thematic analysis where main aims were: (1) facilitator skills, home visits, group support (2) parent-child relationship and parenting (3) parental wellbeing (4) barriers to engagement | -Facilitator rapport and group support were crucial in reducing dropout and increasing program engagement  - Facilitator-client relationship enhanced through home vists  -High program satisfaction  -PLSP addressed barriers to parental engagement: fear, stigma, transportation, distrust, self-esteem  -Enthusiasm, empathy, and openness of program facilitators improved engagement  -Peer support in group sessions improved engagement in a non-judgemental/stigmatizing environment  -Barriers to engagement included: program not appropriate for older children, difficulty understanding, lack of advertisement, repetitive content, attrition of fellow group members  - Rural participants had reduced accessibility to proram  -Poverty, housing instability, social isolation, fear, and stigma decreases service usage  -ChARM model connects parents with other social supports which increases their engagement in community services  -Higher dropout rate for participants with mental health issues |
| *López-Zerón et al., 2021, United States* | Qualitative; semi-structured interviews | 14 parents (13 female) involved with child welfare services | -Cultural adapted parenting training interventions targeting low-income ethnic minority families in child welfare services | Constructionist thematic analysis to determine participant perception and program adaptation to familial needs | -Program improved parents’ ability to interact with child welfare personnel  -Themes identified for their needs: (1) empowerment (2) hope (3) fostering community (4) coping with separation and reintegration (5) personal growth (6) setting limits (7) monitoring/supervision  -Interacting within a group environment was beneficial  -Barrier to engagement: parents commonly experience anger, stress, and disappointment in first sessions of a program; to decrease barriers, program had two introductory sessions to improve engagement  -Simplified materials increased program adherence  -Modify examples, homework, and materials to increase contextual relevance increased engagement  -Cultural and contextual adaptations improved participant engagement |
| *Williams et al., 2022, North Macedonia, Republic of Moldova, and Romania* | Mixed-method | 140 parents (137 female) | -Evaluated the Parenting for Lifelong Health for Youth Children (PLH-YC) program | Factors associated with barriers to engagement (obstacles to engagement scale), factors that increase engagement, premature drop-out, program enrollment, participation rate, parent satisfaction | -Barriers to engagement included: session frequency, timing, lack of childcare  -Factors that increased engagement included: facilitator support, facilitator characteristics, weekly text messages/phone calls, transportation, childcare, sharing lived experiences with other parents  -Factors that increased premature drop-out were: parents of boys, being a victim of intimate partner violence, multiple children, and better well-being  -75% of those enrolled completed the program |
| **Services Targeting Fathers (n=4)** | | | | | |
| *Duggan et al., 2004, United States* | Randomized controlled trial | 643 families | -Hawaii’s Healthy Start Program (HSP) is a home-visiting model for at-risk families  -Target fathers (violent and nonviolent) | Fathers participation in home visiting, program impact on fathers’ parenting involvement, satisfaction among mothers, fathers’ engagement, fathers’ accessibility | -Fathers were less likely to participate if they worked, were physically violent, and consumed alcohol heavily  -For parents who lived together, decreased participation by fathers who were violent (0.18 vs 0.26, p = 0.08)  -For parents who lived apart but saw each other regularly, violent fathers participated more in visits than nonviolent (mean proportion 0.26 vs 0.15, p = 0.07)  -Maternal ratings of fathers’ engagement was 11.4 +/-7.2 (between  “sometimes” and “often”)  -Fathers’ accessibility and engagement significantly decreased over time  -No significant impact on fathers who were violent  -Overall, fathers had less contact with the home visitor than mothers  - Increased accessibility partially mediated program engagement  - Home visitors self-reported more competence working with mothers than fathers |
| *Guterman et al., 2023, United States* | Randomized controlled trial | 204 families | -Dads Matter-HV is a perinatal home visitation program that targets father engagement to reduce physical child abuse risk by both parents  -Mothers and fathers interviewed at 4 months and 12 months post-enrollment | Relational Health Index scale used to assess fathers’ perceptions of their relationship to the home visitor. Assesses (1) engagement (2) authenticity (3) empowerment | -Early engagement with fathers is essential  -92% retention rate at 4 months post-enrollment, 84% retention rate after 12 months post-enrollment  -Child abuse risk reduction after 12 months  -Lowered bidirectional partner abuse  -Fathers who had a stronger relationship with home visitor had a reduced risk of child abuse  -Improved father-home visitor relationship, improved partner support, and predicted lower risk of child abuse at 4 months and 12 months post-enrollment |
| *Veale et al., 2019, Lebanon* | Qualitative study | Focus groups discussions with men (n =130), wives (n = 28), children (n = 17) and families (n=10) | -Evaluates the Engaging Men intervention  -Engaged men to improve family relations, meet psychosocial needs, and promote safety in the community  -Aim to challenge and prevent gender-based violence against girls and women | Participant motivation, experience of participation | -Beneficial outcomes for psychosocial wellbeing, stress management, emotional regulation among men  -Program showed benefits being targeted for men  -Group setting improved socialisation, sense of belonging, expanded social network, and allowed men to discuss topics they otherwise were not likely to  -Relieved psychological distress and pressure  -Increased sense of value among men  -Reduced anger, irritability, and physical punishment at home |
| *Chan, 2004, China* | Quasi-Experimental; one group pretest-posttest | 17 males | -Targets men with two or more reported violent incidents against partners  -Testing the effectiveness of group treatment (2 hours/week x 8 weeks) aimed to increase awareness and decrease violent behavior | Treatment dropout rate over time, attitudes towards group treatment | -Completion rate of all 8 sessions was 82.4% (dropout rate was 17.6%).  -Participants indicated group treatment was both supportive and useful through learning from others and expanding their perspective  -Trust within group increased engagement in later counselling sessions |
| **Services Targeting Children (n=1)** | | | | | |
| *Fairchild and Skewes McFerran, 2019, Australia* | Qualitative; narrative | 15 children ages 8-14 years old | -Targets children experiencing homelessness and/or family violence  -Collaborative music workshop where children worked on group song writing | Examining the perspectives of what music means to children by having them collaborate and create songs about it together | -Music offers hope  -Music provides an escape from the outside world  -Music was identified as a significant personal resource by the children  -Important to evaluate power dynamics during collaboration to provide a safe and encouraging space for all children |
| **Service Provider Perspectives of Facilitators and Barriers to Engagement (n=7)** | | | | | |
| *Bellamy et al., 2024, United States* | Qualitative data collection following randomized controlled trial | 26 home visitors | **-**Dads Matter-HV designed to increase fathers’ engagement in home visitation programs  -Dads Matter-HV aims to reduce barriers to fathers’ engagement | Engagement strategies by home visitors trained in Dads-Matter HV (intervention group) versus services as usual (control group), differences in strategies for fathers, mothers, or both parents | -Early engagement of fathers is essential for ongoing engagement throughout  -Intervention can be provided to both parents conjointly or separately based on familial needs (i.e. work schedules, relationship status)  -Intervention can be provided in-personal or virtually for improved accessibility  -Identified engagement strategies for both parents used: “(1) tell and show parent that they are important; (2) explain the purpose or benefit of the program; (3) make intentional effort to involve parent; (4) address needs; (5) make them feel comfortable and build a relationship; (6) invite parent to participate in activities; (7) leave information for parent; (8) communicate through other parent; (9) offer incentives; (10) provide reminders; (11) make eye contact; (12) parent-centered scheduling; (13) provide encouragement and positive feedback; (14) demonstrate skills and activities; (15) learn parents’ name; (16) provide food”^(p203)^  -Home visitors engaged fathers through external events such as fatherhood programs, family events, and father-daughter dances and inviting mothers to external events such as parenting groups also increased engagement  -Identified engagement strategies for mothers included building rapport and “addressing their needs, particularly their needs for concrete things like diapers, clothing, a birth certificate, information about breast feeding and postpartum issues, and public assistance programs”^(p205)^ |
| *Grönte et al., 2023, Sweden* | Qualitative; case study | 15 social workers; 31 semi-structured interviews total | -Social workers perceptions and strategies used when engaging fathers suspected of inter-partner domestic violence in child protection investigations | Thematic analysis of two main themes when engaging fathers: (1) safety-creating strategies for improved emotional safety (2) strategies to improve communication regarding domestic violence | -Social workers had two main strategies to increase cooperation by fathers: (1) securing an initial bond through: reducing tension, avoiding confrontational interactions, emphasizing support, and affirming the father (2) maintaining the bond while addressing abusive behavior through: negotiating to find a common definition of abuse, and shifting the father’s perspective to improve understanding about domestic violence |
| *Healy and Darlington, 2009, Australia* | Qualitative; case study (vignette) | 28 practitioners in child protection service domains | -Child protection practitioners’ perceptions of the factors in their practice domain, practice approaches, and child and parents’ characteristics that shaped opportunities for family members’ participation in child protection | Determining common themes in principles of participation | -Three core principles of participatory practice were collectively determined by the practitioners: respect, appropriateness, and transparency |
| *Mandara et al., 2023, Australia* | Qualitative | 100 social workers | -From perceptions of social workers  -Determine identification and response of domestic violence among adults and children | Thematic analysis as reported by respondents | -When responding to domestic violence, 1/3 of social workers in sectors outside of domestic violence felt they did not have the skills to provide supportive intervention  -Emphasis on engaging by building trust and collaborating with other agencies for support  -Participants reported approaching adult and child domestic violence differently  -Participants reported not engaging with the perpetrator  -Identified a need for skill development in engaging with the perpetrator |
| *Seekamp et al., 2023, Australia* | Qualitative; semi-structured interviews | 14 professionals in child protective services | -Target professionals perceptions working with children and families in child protective services | Thematic analysis, identified themes include: (1) engaging families in service provision (2) partnering with families | -Practitioner-family relationship, including being genuine and authentic is essential to engage families  -Coercive strategies for engagement led to more challenges engaging families  -Key barrier to engagement identified as judgement  -Professionals who collaborated with others improved engagement with families as it improved the practitioner-family relationship  -Child’s perspective crucial for identifying familial priorities |
| *Svensson et al., 2022, Sweden* | Focus groups | 16 child protection services professionals (support group leaders) | **-**Identified barriers to parental and children engagement in child protection services (CPS) | Thematic analysis to understand barriers to parental/child engagement/participation, identified destigmatizing strategies to improve engagement | -Four themes identified as barriers to participation due to stigma: family secrets – children not being aware of problems or refusing to divulge their situation; parents’ fear of acknowledging harm – parental resistance to their children’s participation due to shame; connection to CPS – parents’ perception of CPS; and scale of shamefulness – domestic violence was one of most difficult for parents to admit  -Identified destigmatizing strategies to reduce barriers:  balancing on defusing the problem and confirming children’s needs; neutralizing participation through procedural strategies – preparing children and parents through initial meetings; creating a sense of ordinariness; disconnecting from CPS – support groups for children appearing separate from CPS; and using deliberate recruitment strategies |
| *Wendt et al., 2020, Australia* | Qualitative; participatory action research | 9 counselors | -Targets men that use abusive or violent behaviours in their relationships  -Perspectives of service providers regarding themes about engaging family members when there is intimate partner violence | Thematic analysis | -Counselors use narrative therapy ideas to encourage curiosity in conversations with couples  -Specific engagement strategies included discussing ethics to explore the effects of power, violence, and fear perpetrated by the male partners  -Important to explore dynamics of power and control in relationships and other possibilities in relationships  - Identified themes represent increased engagement through navigating open conversations and included (1) noticing and curiosity (2) balancing personal assumptions and needs and expectations of each partner (3) focusing on him for her (4) power and control (5) possibilities through exploring alternative actions with the perpetrator |
